# Non-enzymatic glucose sensor with electrodeposited silver/carbon nanotubes composite electrode

**DOI:** 10.1042/BSR20181983

**Published:** 2019-06-25

**Authors:** Syeda Ammara Shabbir, Sana Tariq, M. Gul Bahar Ashiq, Waqar A. Khan

**Affiliations:** 1 Department of Physics, Forman Christian College (A Chartered University), Lahore 54600, Pakistan; 2 Department of Physics,College of Science, Imam Abdulrahman Bin Faisal University, P.O.Box 1982, 31441, Dammam Saudi Arabia. ^2^Basic and Applied Scientific Research Center,Imam Abdulrahman Bin Faisal University, P.O.Box 1982, 31441, Dammam Saudi Arabia; 3 Department of Mechanical Engineering, College of Engineering, Prince Mohammad Bin Fahd University, Al Khobar 31952, Kingdom of Saudi Arabia

**Keywords:** carbon nanotubes, glucose sensor, nanocomposites, nonenzymatic, silver nanoparticles

## Abstract

Diabetes mellitus is a debilitating disease that affects each and every organ of human body. Hence it is important to continuously monitor the glucose level throughout the day and night. Glucose sensors are in great demand due to a rapid increase in diabetic community. A strategy has been implemented here to fabricate silver nanoparticles (AgNPs) with the support of functionalized carbon nanotubes (f-CNTs). Silver/carbon nanotubes (Ag/CNTs) nanocomposite electrode have been prepared by electrochemical process on Fluorine doped tin oxide (FTO) glass, by varying silver (Ag) concentrations for non-enzymatic glucose sensor. The variable Ag concentration in the morphology of Ag/CNTs nanocomposite has influenced the electrical conductivity, oxidation and reduction potential and electrochemical activity of glucose. Highest current density and good electrocatalytic activity for electrodes are obtained at 70 mM concentration of silver in Ag/CNTs composite. The present study indicates that the Ag/CNTs electrode is a possible substitute of the expensive glassy carbon electrode for enzyme-free glucose sensors.

## Introduction

Diabetes mellitus being a major health problem affects millions of people across the world. One of the prime causes of diabetes mellitus is obesity as it has a direct relation with insulin resistance, which ultimately worsens the disease. Human body requires the right amount of insulin to be released, therefore it is very essential to measure the glucose level throughout day and night. Continuous glucose monitoring (CGM) plays a very important role in fulfilling this objective [1]. Timely monitoring of blood sugar levels can ease the complications. Commercially portable glucose sensors, which quantitate blood glucose using finger pricks, fail to measure rapid and elevated glucose changes. These glucose sensors are based on enzymes and have several drawbacks, consisting in high cost and low stability, due to inherent properties of enzymes. CGM sensors are intended to solve this problem using non-enzymatic nanosensors. CGM devices utilize electrochemical detection of glucose which facilitates quantitation of glucose with high sensitivity and selectivity. Unfortunately, these sensors provide unavoidable drawbacks as being slow response time, irreproducibility and less stability over time [2]. Therefore, development and research of nanosensors for diabetes management is an important area nowadays with rapid progress.

Our body contains different organic fluids such as blood serum and urine, these fluids consist of multiplex structures and composite matrix. The electrochemical measurement of glucose becomes a strenuous task as there is a probability of these complex structures to restrain and interfere while measuring glucose. Therefore a sensitive approach must be brought into interest which makes a selective glucose measurement. Enzymes are expensive and have complicated immobilization techniques which makes it hard to restrict enzymes’ mobility to a fixed space. Enzyme-modified electrodes are incapable of measuring continuous blood glucose level [3–7]. The fourth generation of biosensors, namely the non-enzymatic biosensors have therefore gained vast research attention for glucose sensing keeping in view the desired properties they possess such as good sensitivity and selectivity, high conductivity, stability and reproducibility, low detection limit and cost effectiveness [8–10].

The most important outcome in this research area to date is that highly electroactive surface area plays an important role in glucose electroxidation. Different nanoparticles can be used for enzyme-free glucose sensing, but the nanoparticles of transition metals such as copper (CuNPs), nickel (NiNPs), gold (AuNPs), platinum (PtNPs) and silver (AgNPs) nanoparticles are preferred due to their property to rapidly accept an electron. It is crucial to make the best pick while selecting the nanoparticles for the fabrication of non-enzymatic sensor. The literature survey reveals the fact about some of the disadvantages of these metal nanoparticles. Copper nanoparticles [11] are unstable under atmospheric conditions; nickel nanoparticles [12] have toxic effect on the immune system, gold nanoparticles [13] also show some toxic effects, have less efficiency and require a stabilizing agent to lessen the rate of agglomeration and the high cost of platinum plays a major selection barrier. In contrast, silver nanoparticles (AgNPs) tend to be more stable, non-toxic, biocompatible, antibacterial, cost effective and show high conductivity and good electrocatalytic activity [14]. Carbon nanotubes as a supporting electrode material play an important role in catalytic activities due to high surface to volume ratio [15–18].

In this research project, electrochemical process was used to fabricate non-enzymatic sensors for measuring glucose levels in tissue fluids. The nanohybrid electrodes were synthesized by depositing functionalized CNTs (f-CNTs) and AgNPs) on fluorine doped tin oxide (FTO) glass. The morphological characterization of the nanocomposite electrode (Ag/CNTs) showed the presence of CNTs and Ag nanoparticles. Variations in the concentrations of AgNPs were made to observe different parameters like average particle size, electrocatalytic activity, grain size, sensitivity and selectivity of glucose toward the electrodes with different concentrations. The fabricated nanocomposite electrode offers high sensitivity toward glucose and electrocatalytic activity. Maximum electrocatalytic activity was observed to be for 70 mM concentration of Ag/CNTs as compared with other concentrations. It also shows highly electroactive and sensitive electrode for glucose detection by providing an economical route.

## Experimental

### Materials

The covalently bonded COOH f-CNTs were further functionalized with sodium dodecyl sulfate (SDS) in a deionized (DI) water solution. The other chemicals used in experiment were silver nitrate (AgNO_3_), sulphuric acid (H_2_SO_4_), sodium hydroxide (NaOH) and glucose. Glucose was used without any further purification. All the solutions were prepared using DI water.

### Synthesis of Ag/CNTs electrode

The precursor solution for CNTs was prepared by dissolving 50 mg of COOH f-CNTs in 0.075 g of SDS and 50 ml DI water. For a stable and homogeneous dispersion of SDS f-CNTs the solution was sonicated for an hour and further centrifuged at 8000 rpm for 30 min. This substantial dispersion was spin-coated on FTO substrate at 3000 rpm for 80 s. The synthesized CNTs electrodes were dried at room temperature. The electrodes were then employed for electrodeposition of Silver (Ag) through cyclic voltammetry. A one compartment cell comprising a setup of three electrodes in an electrolyte solution of 0.1–1 M AgNO_3_ and 0.2 M H_2_SO_4_ was used to accomplish the electrodeposition of Ag.

To observe the change in the electrocatalytic activity the concentration of AgNO_3_ was varied for the fabrication of four samples of Ag/CNTs. The scan rate was selected to be at 100 mV/s and the potential window to be at −0.5 to 1.0 V. The number of cycles during cyclic voltammetry was fixed to be 20 to achieve the high reproducibility and sensitivity of the electrodes. The highly uniform and homogeneous electrodeposition of Ag was conducted on spin-coated SDS f-CNTs electrodes. The same process was repeated for different concentrations of AgNO_3_. The fabricated Ag/CNTs nanocomposite electrodes were stored at room temperature.

### Characterization

XRD analysis of Ag/CNTs electrodes were performed through XRD 2001 Bruker diffractometer. XRD was used to determine the composition of the electrodes. The surface morphology of nanocomposite Ag/CNTs electrodes were analyzed by SEM; JSM-6480 LV. From SEM graphs, particle size of approximately 20 particles was calculated using ImageJ software. Histograms of these variable particle sizes plotted the number of particles versus the particle size. Gaussian curve was taken for the respective histogram to know about the average particle size. The peak or amplitude of Gaussian curve tells us the average particle size within a specific sample. The electrochemical catalytic properties and cyclic voltammetry were performed through Princeton 263 A work station for the evaluation of the oxidation-reduction (redox) behavior of AgNPs on the surface of Ag/CNTs electrodes and to deduce a precise verification for the formation of AgNPs.

## Results and discussion

**Figure 1:**
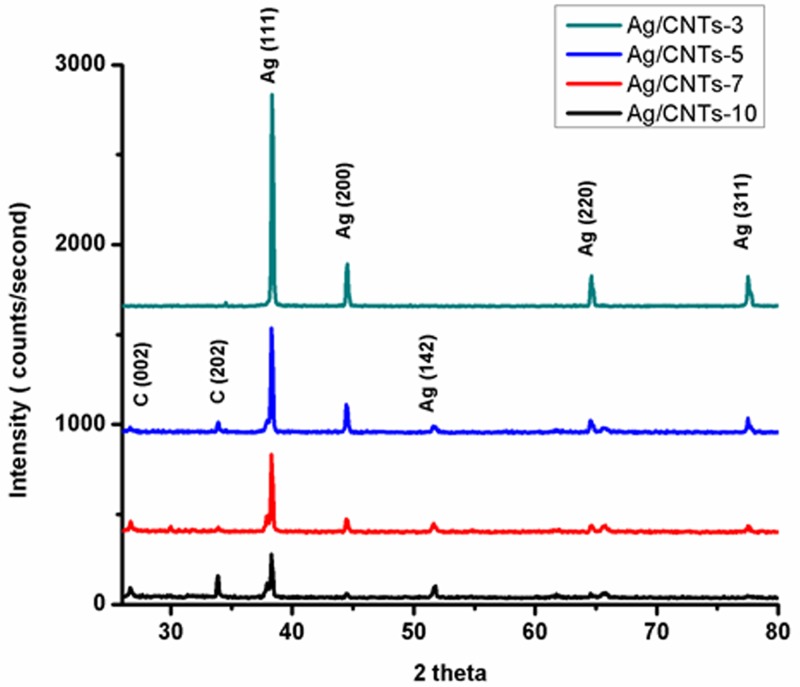
XRD pattern of Ag/CNTs-3, Ag/CNTs-5, Ag/CNTs-7 and Ag/CNTs-10 electrodes

The XRD pattern in Figure 1 corresponds to Ag/CNTs electrodes with different concentrations of silver i.e. 30 mM for Ag/CNTs-3, 50 mM for Ag/CNTs-5, 70 mM for Ag/CNTs-7 and 100 mM for Ag/CNTs-10, respectively. The diffraction peak at 26° and 33.9° is of CNTs corresponding to C (111) and C (202) planes. These diffraction planes correspond to the graphite having cylindrical structure with inter-layered spacing. The 2θ peaks at 38.34°, 44°, 51°, 64° and 77° correspond to Ag (111), Ag (200), Ag (142), Ag (220) and Ag (311) planes, respectively. These results demonstrate that the AgNPs have been electrodeposited on the SDS f-CNTs coated FTO electrodes successfully [18–20]. With the higher concentration of silver, there is an eminent increase in the peak strength corresponding to Ag (111). This shows an increase in crystallinity of Ag planes due to increase in grain size. As the grain size increases, there will be more chances of planes being in symmetry resulting in increased crystallinity and constructive interference. However at higher concentrations of silver deposition, the peaks corresponding to CNTs start getting invisible. This could be attributed to the changes caused on the surface of nanotubes by adding defects while making modification to generate a composite material, linking nanoparticles to carbon nanotubes. This fabricated hybrid material also changes CNTs hybridization from sp^2^ to sp^3^ that eventually breaks the periodicity of 1-D CNTs [21].

The change in grain size of AgNPs changes with varying concentrations of Ag. The quantitative measurement of this change can be calculated by applying Scherrer formula to XRD data.

**Scherrer formula:**

D  =  kλ /Bcosθ

Where,

D = is the grain size

k = the shape factor = 0.9

λ = X-rays wavelength in XRD = 1.54 Å

B = Full width at half maximum

θ = is in radian [22,23]

Table 1 below gives us the information about the change in grain size. The grain size increases with the increase in the concentration of AgNPs. The following concentrations of Ag i.e. 30, 50, 70 and 100 mM correspond to the average grain size of 24.82, 28.88, 32.20 and 39.68 nm. The surface to volume ratio of nanoparticles decreases as the grain size increases with the increase in Ag concentrations.

**Table 1:** Average grain size of Ag/CNTs for different concentrations of silver

**Sample 1**	**2θ**	**Hkl**	**Grain size**
Ag/CNTs-3	38	Ag (111)	42.01
Ag/CNTs-3	44	Ag (200)	14.85
Ag/CNTs-3	51	Ag (142)	17.60
**Average grain size = 24.82 nm**			
**Sample 2**	**2θ**	**Hkl**	**Grain size**
Ag/CNTs-5	38.3	Ag (111)	84.07
Ag/CNTs-5	44	Ag (200)	28.62
Ag/CNTs-5	51	Ag (142)	17.60
Ag/CNTs-5	64	Ag (220)	18.83
Ag/CNTs-5	77	Ag (311)	20.29
**Average grain size = 28.88 nm**			
**Sample 3**	**2θ**	**Hkl**	**Grain size**
Ag/CNTs-7	38	Ag (111)	42.01
Ag/CNTs-7	51	Ag (142)	29.33
Ag/CNTs-7	44	Ag (200)	28.56
Ag/CNTs-7	64	Ag (220)	23.48
Ag/CNTs-7	77	Ag (311)	25.46
**Average grain size = 32.20 nm**			
**Sample 4**	**2θ**	**Hkl**	**Grain size**
Ag/CNTs-10	38	Ag (111)	42.01
Ag/CNTs-10	44	Ag (200)	28.61
Ag/CNTs-10	64	Ag (220)	62.65
Ag/CNTs-10	77	Ag (311)	25.46
**Average grain size = 39.68 nm**			

**Figure 2:**
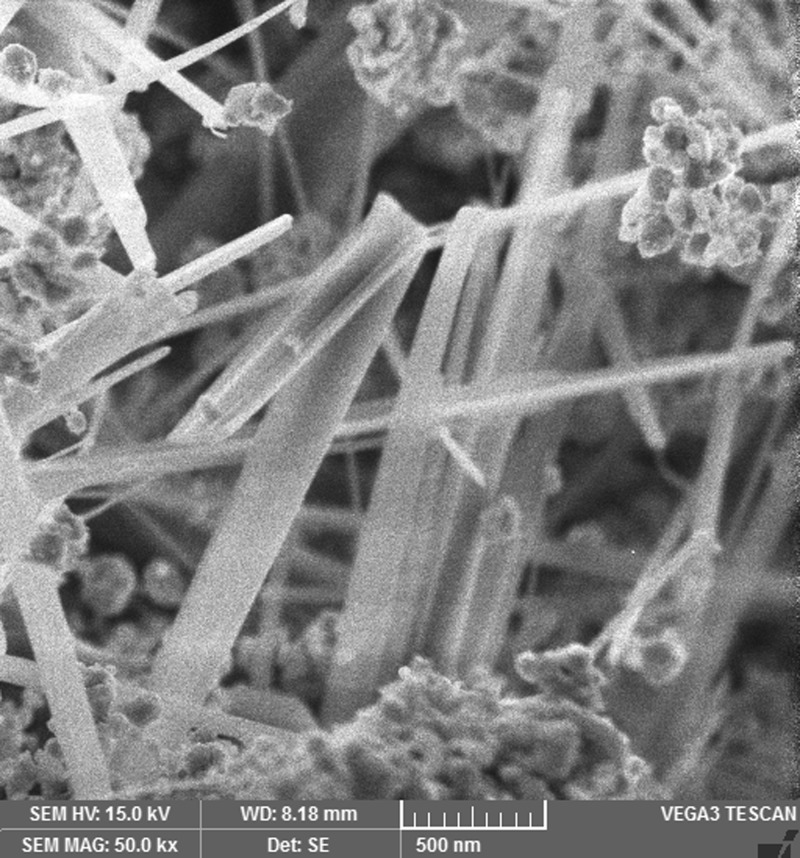
SEM image of Ag/CNTs-3 electrode

The images shown in Figures 2,4,6 and 8 are acquired by SEM analysis of Ag/CNTs-3, Ag/CNTs-5, Ag/CNTs-7 and Ag/CNTs-10 electrodes. The analysis about the surface morphology confirmed the spherical shape of AgNPs and tubular shape of CNTs. The increase in the concentration of silver is directly related to the increase in the number of AgNPs. Moreover the AgNPs have also increased in size due to agglomeration of smaller sized nanoparticles. Some adjacent particles combine during aggregation, as the size proliferates. Histograms were plotted between the particle size (nm) and the number of particles to calculate the average particle size for all the samples having different concentrations of Ag using the data from SEM analysis.

**Figure 3:**
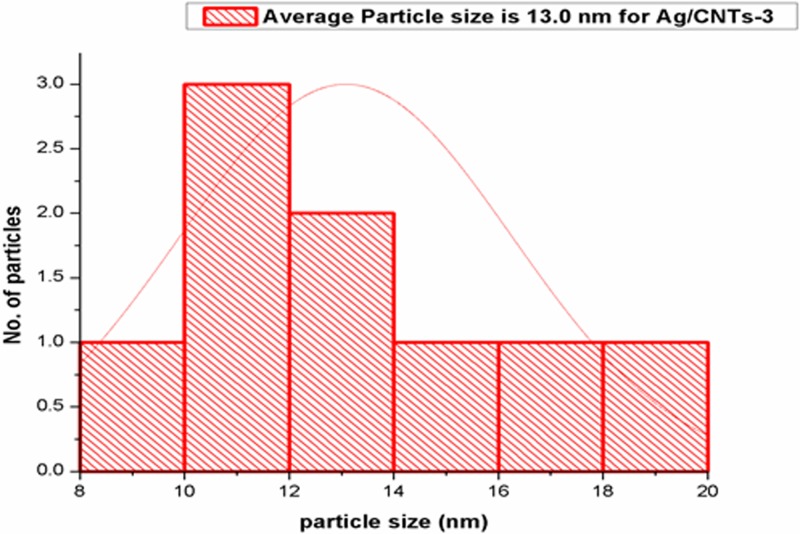
Average particle size calculation for Ag/CNTs-3 electrode

Figures 3,5,7 and 9 depict the histogram associated with varying concentrations of AgNPs with certain particle size. The average particle size was calculated by applying Gaussian curve fit. The average particle size for Ag/CNTs-3, Ag/CNTs-5, Ag/CNTs-7 and Ag/CNTs-10 was calculated to be 13.0, 27.9, 53.8 and 70.0 nm respectively. The increase in particle size with increase in amount of silver during electrodeposition has been observed.

**Figure 4:**
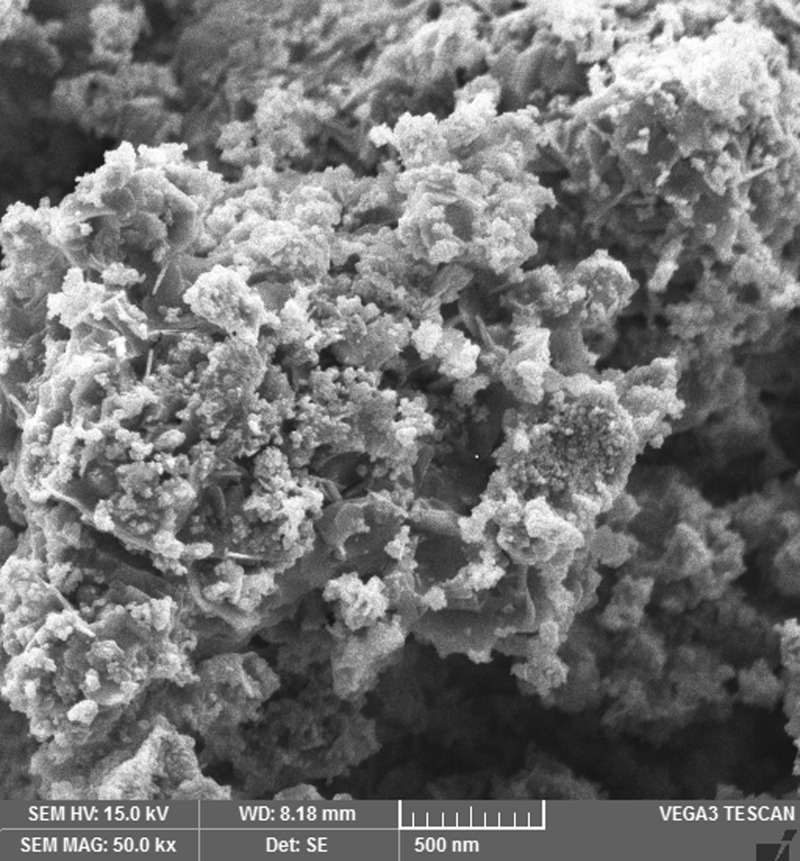
SEM image for Ag/CNTs-5 electrode

**Figure 5:**
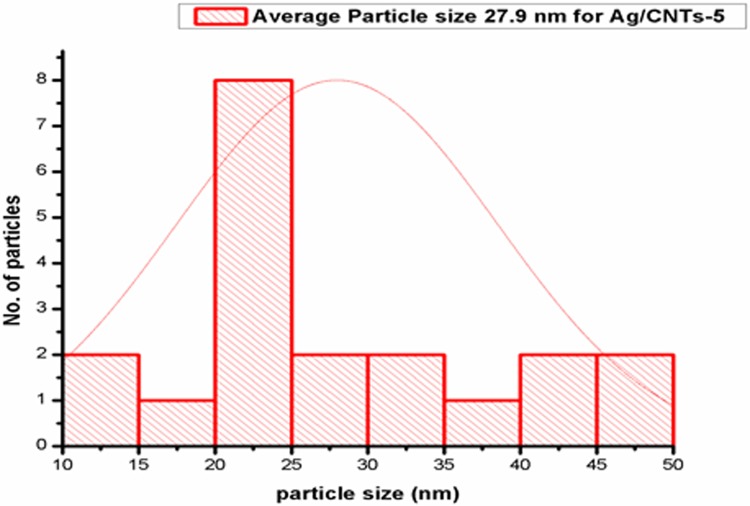
Average particle size calculation for Ag/CNTs-5 electrode

**Figure 6:**
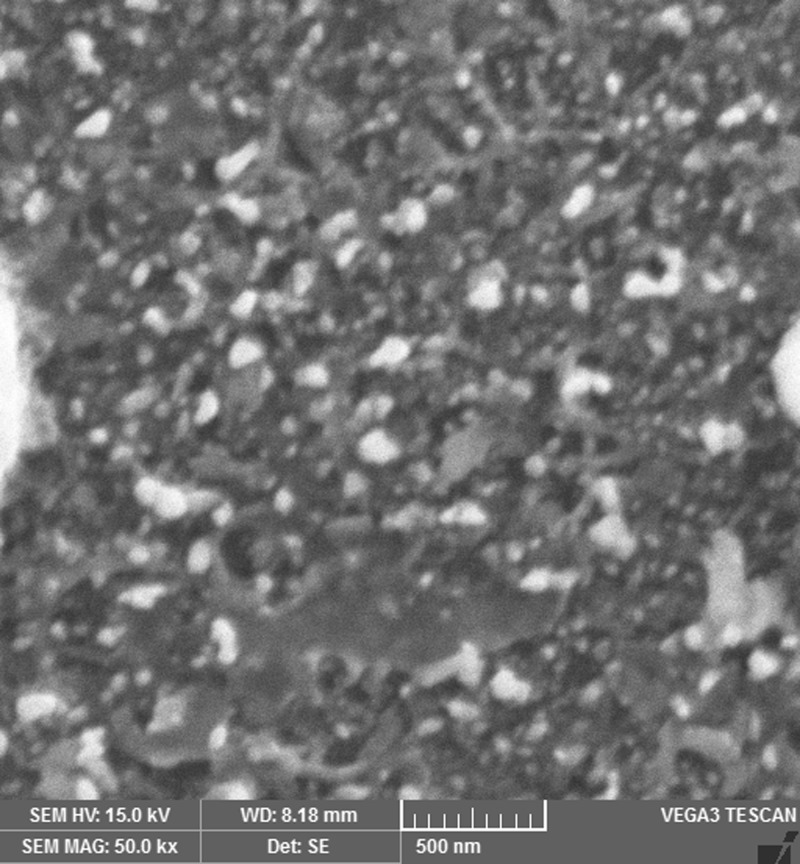
SEM image for Ag/CNTs-7 electrode

**Figure 7:**
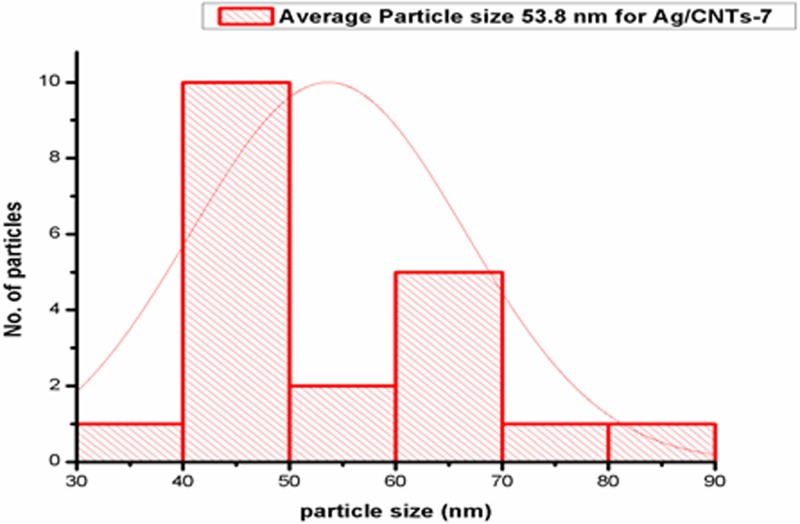
Average particle size calculation for Ag/CNTs-7 electrode

**Figure 8:**
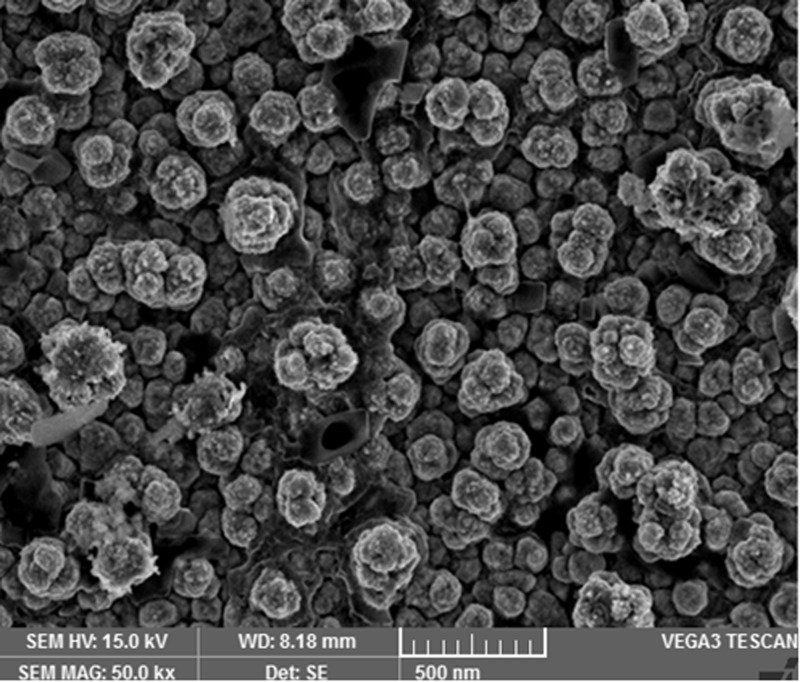
SEM image for Ag/CNTs-10 electrode

**Figure 9:**
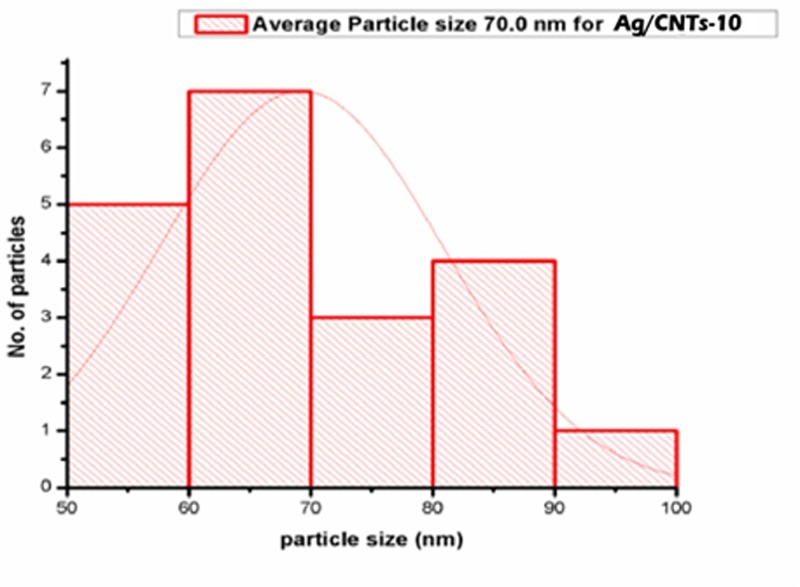
Average particle size calculation for Ag/CNTs-10 electrode

Table 2 represents the change in average particle size with increase in the concentration of Ag.

**Table 2:** Average particle size of Ag/CNTs electrodes for different concentrations of silver

Silver concentrations	Average particle size (nm)
Ag/CNTs-3	13.0
Ag/CNTs-5	27.9
Ag/CNTs-7	53.8
Ag/CNTs-10	70.0

**Figure 10:**
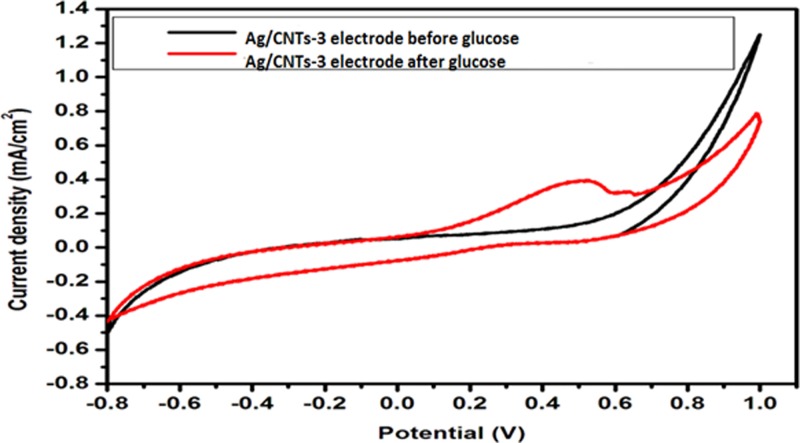
Cyclic voltammogram of glucose oxidation for Ag/CNTs-3 electrode

**Figure 11:**
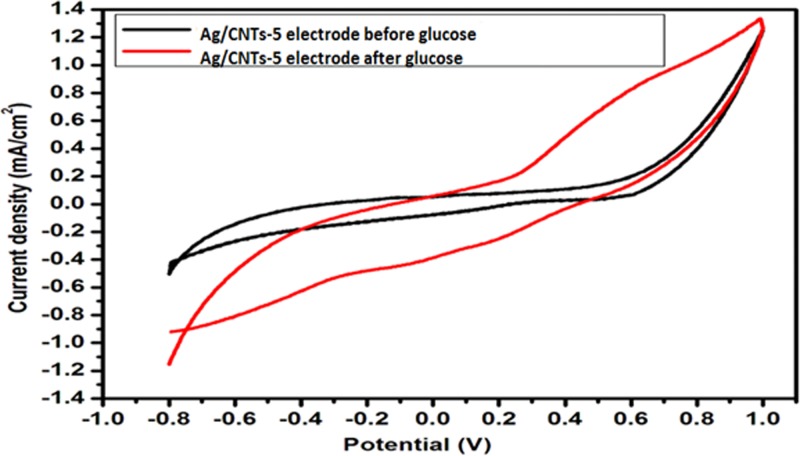
Cyclic voltammogram of glucose oxidation for Ag/CNTs-5 electrode

**Figure 12:**
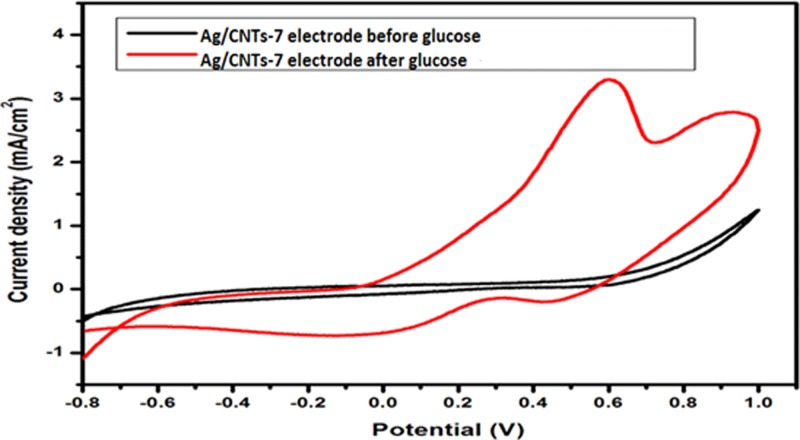
Cyclic voltammogram of glucose oxidation for Ag/CNTs-7 electrode

**Figure 13:**
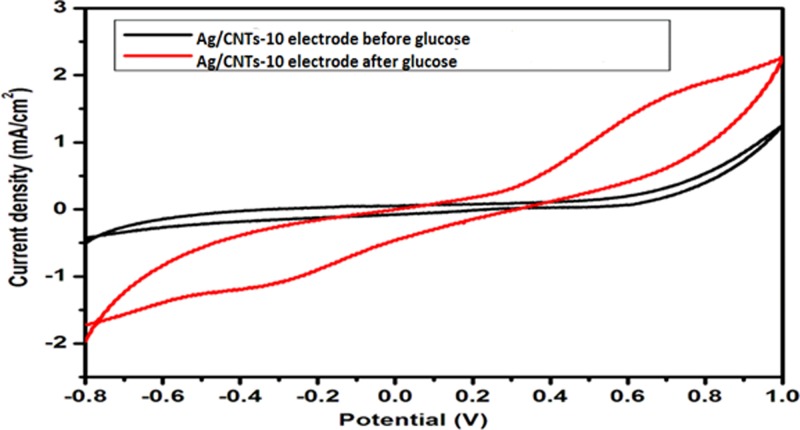
Cyclic voltammogram of glucose oxidation for Ag/CNTs-10 electrode

Figures 10–13 given below show us the cyclic voltammograms for all the samples, i.e. Ag/CNTs-3, Ag/CNTs-5, Ag/CNTs-7 and Ag/CNTs-10. Cyclic voltammetry was use to establish the electrocatalytic response of these electrodes toward glucose oxidation. The potential range was specified to be 1.0 to −0.8 V at the scan rate of 20 mV/s. The data in black represent the cyclic voltammogram with only 0.1 M NaOH alkaline solution present as an electrolyte in the absence of glucose and the data in red demonstrate the cyclic voltammogram in the presence of 0.1 M NaOH + 0.5 mM glucose. Addition of NaOH as an electrolyte facilitates the ionic conductivity which helps glucose to move swiftly toward the electrode [24]. In the absence of glucose no peak for reduction and oxidation has been observed. However, a drastic and notable increase is observed in the redox peak currents after the addition of glucose in the electrolyte. This indicates to the fact that AgNPs are highy electroactive toward glucose while carbon nanotubes provide the large surface area and conductivity to enhance the electron transfer toward electrode. We can observe from the following Figures 10–13, that the anodic peak current related to the 30, 50, 70 and 100 mM concentrations of Ag are 0.4, 0.8, 3.3 and 1.3 mA/cm^2^. The highest anodic peak current for glucose oxidation is obtained when the concentration of Ag is 70 mM. Table 3 shows the increase in electrocatalytic activity and reached maximum at 70 mM concentration of Ag but further decreased at higher concentration of 100 mM. This increment in electrocatalytic activity till 70 mM is due to the fact that the increase in the rate of loading increases the AgNPs size that are available for adsorption. Now the decrement in electrocatalytic activity at the higher concentration of 100 mM of Ag is because the nanoparticles start agglomerating with the increase in AgNPs size resulting in the decrease in active sites for electrocatalytic activity. From the data achieved from cyclic voltammetry the maximum electrocatalytic activity has been obtained at 70 mM concentration of Ag/CNTs [25,26]. Table 3 given below shows us the anodic peak current corresponding to different concentrations of Ag.

**Table 3:** Measurement of anodic peak current for different concentrations of silver

Concentration	Potential (V)	Anodic peak current Ia (mA/cm^2^)
Ag/CNTs-3	0.5	0.4
Ag/CNTs-5	0.6	0.8
Ag/CNTs-7	0.6	3.3
Ag/CNTs-10	0.7	1.3

**Figure 14:**
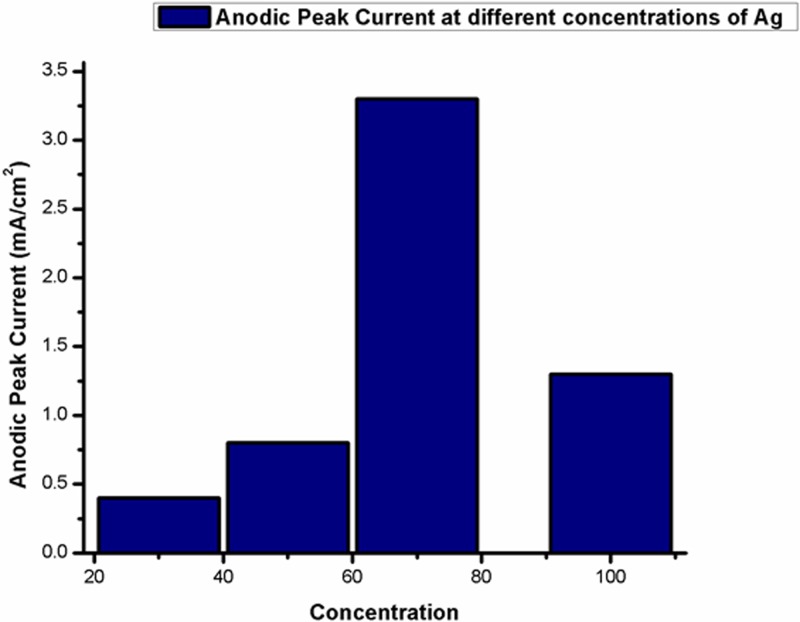
Graphical representation of variations of anodic peak current

Figure 14 shows the graphical demonstration of Table 3, we can analyze from the graph that 3.3 mA/cm^2^ is the maximum anodic peak current obtained at 70 mM concentration of Ag and then it gradually decreases.

**Figure 15:**
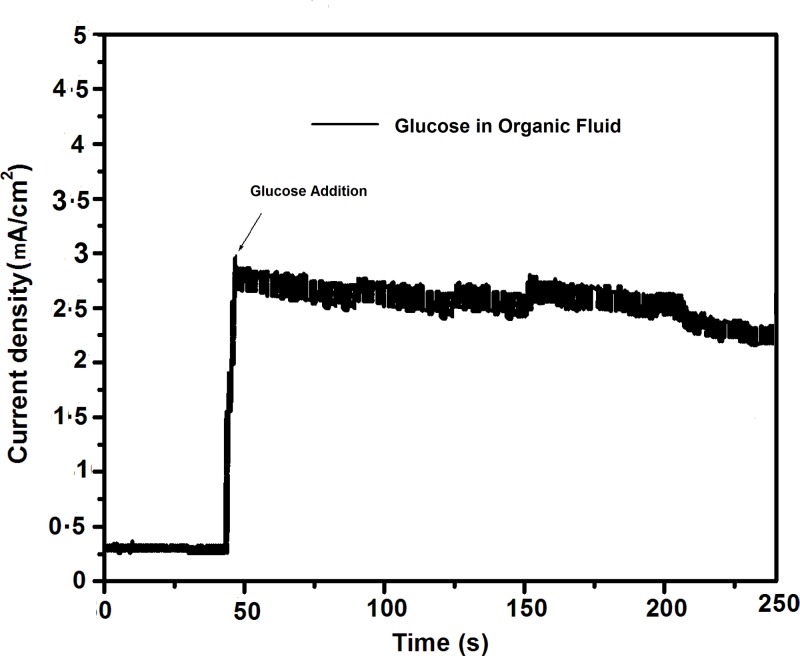
Amperometric measurements for glucose detection in organic fluid

To explore the practical applications of non-enzymatic electrodes, several testings on human blood serum samples have been performed for glucose measurements in alkaline conditions [27–30]. Herein, glucose concentration in organic fluid of orange juice has been analyzed using Ag/CNTs-7 electrode, for which maximum anodic peak current was measured for glucose oxidation at 0.575 V. The glucose detection response was determined by taking 1 ml (3 mM) orange juice in 20 ml (0.1 M) NaOH. Amperometric response as shown in Figure 15 was detected at applied potential of 0.575 V upon addition of orange juice. Maximum current was detected to be 2.9 mA/cm^2^. The orange juice sample showed less current response of glucose detection due to presence of several types of molecules available in orange juice. Therefore, fabricated Ag/CNTs/FTO electrodes hold potential for practical detection of glucose in organic fluid samples.

These nanoscale sensors have showed improved potential for direct CGM and thus can be effectively used for better patient quality of life. In order to achieve the ultimate goal of accurate and long-term CGM, these fabricated nanosensors can be compared with commercially available sensors to justify the cost and effort required for manufacturing of nanosensors in comparison with the standard available sensors.

## Conclusion

The fabrication of Ag/CNT-based nanocomposite electrode for non-enzymatic glucose sensor was performed by electrochemical process. The SDS and COOH f-CNTs solution offered immensely dispersed solution for uniform and homogeneous spin-coating of CNTs on the FTO electrodes. Cyclic voltammetry was used to electrodeposit AgNPs of varying concentrations on the fabricated CNTs electrode. The CNT-coated electrode provides a support system to the AgNPs for the highly electroactive surface area. The results demonstrated by XRD confirmed the electrodeposited AgNPs on carbon nanotubes. SEM analysis confirmed the spherical morphology of AgNPs and tubular structure of Carbon nanotubes. The cyclic voltammograms showed that these nanohybrid electrodes (Ag/CNTs) with different concentrations of AgNPs offer good electrocatalytic activity toward glucose detection with cost effective materials. The increase in anodic peak current was observed with increase in concentration of AgNPs. The optimized value of Ag concentration for maximum electroactivity was found to be 70 mM. Amperometric response of Ag/CNTs electrode toward organic fluid (Orange juice) shows that these electrodes can be used in industries for practical and clinical applications as well. These nanocomposite electrodes (Ag/CNTs) can be utilised as the possible substitute to the expensive electrodes being used for non-enzymatic glucose sensors and will provide high sensitivity for real blood samples during continuous glucose measurements.

## Abbreviations

AgNP: silver nanoparticle
Ag/CNT: silver/carbon nanotube
CGM: continuous glucose monitoring
DI: deionized
FTO: fluorine doped tin oxide
f-CNT: functionalized CNT
redox: oxidation-reduction
SDS: sodium dodecyl sulfate
SEM: Scanning electron microscope
XRD: X-rays diffraction
